# Perceived Positions Determine Crowding

**DOI:** 10.1371/journal.pone.0019796

**Published:** 2011-05-24

**Authors:** Gerrit W. Maus, Jason Fischer, David Whitney

**Affiliations:** 1 Department of Psychology, University of California, Berkeley, California, United States of America; 2 Center for Mind and Brain, University of California Davis, Davis, California, United States of America; Istituto di Neuroscienze, Italy

## Abstract

Crowding is a fundamental bottleneck in object recognition. In crowding, an object in the periphery becomes unrecognizable when surrounded by clutter or distractor objects. Crowding depends on the positions of target and distractors, both their eccentricity and their relative spacing. In all previous studies, position has been expressed in terms of retinal position. However, in a number of situations retinal and perceived positions can be dissociated. Does retinal or perceived position determine the magnitude of crowding? Here observers performed an orientation judgment on a target Gabor patch surrounded by distractors that drifted toward or away from the target, causing an illusory motion-induced position shift. Distractors in identical physical positions led to worse performance when they drifted towards the target (appearing closer) versus away from the target (appearing further). This difference in crowding corresponded to the difference in perceived positions. Further, the perceptual mislocalization was necessary for the change in crowding, and both the mislocalization and crowding scaled with drift speed. The results show that crowding occurs after perceived positions have been assigned by the visual system. Crowding does not operate in a purely retinal coordinate system; perceived positions need to be taken into account.

## Introduction

**Figure 1:**
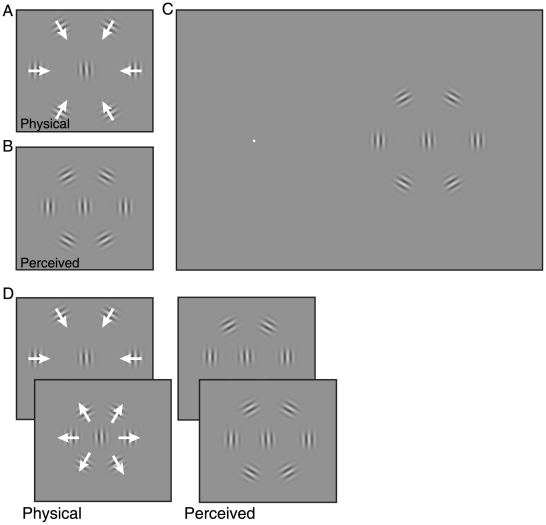
Stimuli. **A–B** The positions of drifting Gabor patterns are perceived as shifted in the direction of internal motion, although their contrast envelope remains stationary [4]–[6]. Physical and perceived positions are dissociated. **C** The main stimulus used in the present study. A tilted Gabor target is presented at 14 degrees eccentricity to the left or right of the fixation point. Surrounding the target are six distractor Gabors arranged in a hexagonal ring, oriented such that they can be drifted either towards or away from the target. **D** In each trial of Experiment 1A, the stimulus was presented twice. In the second presentation interval distractors drifted in the opposite direction of the first interval, and the physical position was changed. Participants judged in which interval the distractors appeared as spaced wider. In the example shown, although the physical spacing in the first (inward drifting) presentation is much larger than in the second (outward drifting) presentation, the stimuli are perceived in identical positions.

Crowding refers to the phenomenon that objects in the visual periphery are harder to discriminate when they are surrounded by other objects. Crowding is not to be confused with the normal decrease of acuity in the visual periphery or with ordinary masking [1], [2]. The severity of crowding depends on the spacing between objects and their position in the visual field. Bouma's rule states that when objects are spaced closer than about half their eccentricity, crowding is experienced [3]. The *positions* of objects in the visual field determine the amount of crowding, both the absolute position (eccentricity) of the target object and the relative positions (spacing) of the flankers to the target. Traditionally, *position* in the context of crowding has been interpreted as the position of an object's image on the retina. In many situations, especially in the static and artificial scenes used in typical crowding experiments, retinal position and perceived position of an object match up well. However, there are some well-documented visual illusions in which retinal and perceived positions of an object can be dissociated. Some particularly powerful examples of these illusions occur when parts of the visual field or the object itself are moving [4]–[10]. For example, a drifting Gabor stimulus (a drifting grating windowed by a stationary Gaussian contrast envelope) appears shifted in the direction of the underlying grating's motion, although the retinal position of the Gabor pattern as a whole remains unchanged [6] (Fig. 1A–B).

In the present study we aimed to determine whether crowding is based on the physical positions of objects on the retina or on their perceived positions. Using the DeValois illusion described above, we systematically manipulated the perceived positions of flanking Gabor gratings around a target grating independently of their retinal positions. We measured observers' abilities to discriminate the orientation of the target dependent on the perceived or physical target–distractor distance. Our findings show that perceived positions determine crowding.

## Results

**Figure 2:**
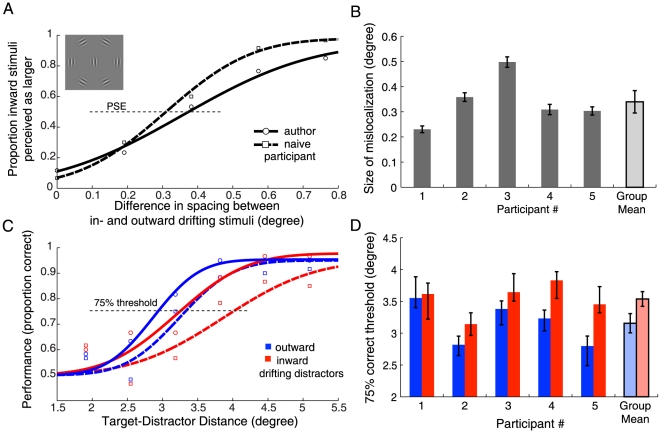
Results from Experiment 1. **A** Raw data and psychometric function fits for one author (circles, solid line) and one naïve participant (squares, dashed line) from Experiment 1A. The x-axis denotes the difference in spacing between inward and outward drifting stimuli; positive values mean that spacing for inward was larger than for outward drift. The y-axis denotes the proportion of trials in which the spacing of inward drifting stimuli was perceived as larger than the spacing for outward drifting stimuli. Points of subjective equality (PSEs) are defined as the point where fitted functions cross 50% responses (thin dotted line). **B** PSEs from psychometric function fits (and bootstrapped 95%-confidence intervals [44]) for all five participants of Experiment 1A. The right-most bar shows the mean PSE of the group (and between-participant standard error of the mean). **C** Raw data and psychometric function fits from Experiment 1B for the same two participants as in A. The proportion of correct responses on the orientation discrimination of the target is plotted against the center-to-center target-distractor distance. Outward drift of distractors (away from the target) is shown in blue, inward drift (towards the target) in red. The red curves are shifted to the right relative to the blue curves, meaning that the distractors drifting towards the target had to be physically further from the target to achieve equivalent performance. **D** 75%-correct thresholds from psychometric function fits (and confidence intervals) for all participants in Experiment 1B. The rightmost bars show mean thresholds (and between-participant standard errors). Outward drifting distractors (blue) led to significantly lower thresholds than inward drifting distractors (red).

First, we measured the size of the motion-induced mislocalization illusion with our particular stimulus display (Fig. 1C). In each trial of Experiment 1A we presented a “ring” of Gabor patches in a hexagonal arrangement, drifting in opposite directions in two sequential intervals, and at the same time varied their physical positions (see Materials and Methods, Fig. 1D). Participants judged the perceived position shift between the first and the second presentation interval. By fitting psychometric functions we estimated the required change in physical position to null the perceived position shift of Gabors drifting in opposite directions. Figure 2A shows data for one author and one naïve participant; individual points of subjective equality (PSEs) for the group are shown in Figure 2B. All participants misperceived the position of drifted Gabors as shifted in the direction of drift motion. The mean size of the illusory position shift across all five participants was 0.34 degrees (S.E.M = 0.04 degrees; two-tailed one-sample t-test *t*(4) = 7.64, *p* = 0.002).

Next, we assessed how the perceived position shift caused by the motion in the distractors affected discrimination performance on the central target. In Experiment 1B participants performed a two-alternative forced-choice (2AFC) orientation discrimination judgment on the central target grating. We varied the physical target–distractor distance and fitted independent psychometric functions for inward and outward moving distractors. Results from two observers are shown in Figure 2C. Performance was generally better when the distractors drifted away from the target, and were thus perceived as spaced wider. Performance thresholds (75% correct) for all participants are shown in Figure 2D. All individual participants showed lower thresholds for distractor movement away from the target. The mean difference in physical position between inward and outward moving distractors that led to threshold performance was 0.41 degrees (S.E.M. = 0.11 degrees; paired t-test *t*(4) = 3.46, *p* = 0.026). Crowding was stronger for distractors drifting towards the target and weaker for distractors drifting away from the target. The difference in crowding between motion directions was equivalent to that caused by a physical change of distractor position of 0.41 degrees, which is comparable to the size of the perceived mislocalization of 0.34 degrees from Experiment 1A.

### Necessity of the perceptual position shift for changes in crowding

**Figure 3:**
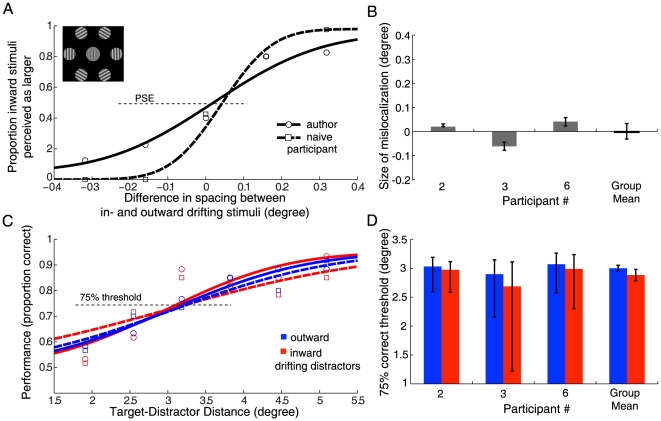
Results from Experiment 2. All data is presented analogously to Figure 2 (Experiment 1). **A** Raw data and psychometric function fits for one author (circles, solid line) and one naïve participant (squares, dashed line) from Experiment 2A. The inset shows the changes to the stimulus (hard apertures for the distractors and lower background luminance). All other aspects of the stimuli and task were identical to Experiment 1. **B** PSEs (and confidence intervals) for all three participants in Experiment 2A. The rightmost bar shows the mean mislocalization effect for the group (and standard error). The stimulus manipulations abolished the motion-induced mislocalization effect. **C** Raw data and psychometric function fits from the crowding experiment (Experiment 2B). There is no clear separation of psychometric functions between inward and outward drifting distractors. **D** 75% correct thresholds (with confidence intervals) for all participants and group means (with standard errors). There was no significant difference in crowding for distractors of opposing motion directions.

To address the possibility that differences in crowding in Experiment 1B were not due to the perceived position shift of the distractors, but due to some other aspect of motion in the stimulus, we repeated Experiment 1 with modified stimuli. In Experiment 2 the distractors had hard apertures instead of Gaussian contrast envelopes, and the background luminance was reduced. Both manipulations are known to reduce the motion-induced mislocalization of drifting gratings [11]–[13]. Analogously to Experiment 1, Experiment 2A measured differences in localization of gratings drifting in opposite directions. Figure 3A shows data for two observers; group results are shown in Figure 3B. The mean perceived mislocalization was 0.00 degrees (S.E.M = 0.03 degrees; *t*(2) = 0.42, *p* = 0.99). This confirms that the stimulus manipulations—introducing a darker background and hard apertures for the distractors—abolished the motion-induced mislocalization.

Experiment 2B assessed whether the difference in motion direction of the distractors per se—without a perceptual mislocalization—has any effect on crowding of the central target. Results of the orientation discrimination task are shown in Figure 3C and D. All three observers showed equivalent performance for distractors moving towards and away from the target. The mean difference was −0.12 degrees (S.E.M = 0.05 degrees; *t*(2) = 0.90, *p* = 0.14). In effect, crowding was unchanged for distractors of opposite motion directions.

**Figure 4:**
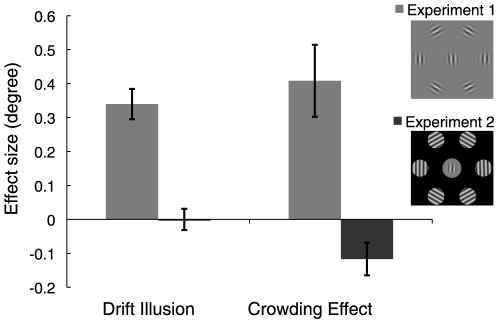
Mislocalization and crowding effects. Mean effect sizes (and standard errors) for Experiments 1 and 2. Stimulus insets show the changes to the stimuli. In Experiment 1 participants mislocalized Gabors in the direction of motion by ∼0.34 degrees and experienced crowding for different motion directions, as if the stimuli were physically shifted by ∼0.41 degrees (light grey bars). In Experiment 2 there was no motion-induced mislocalization and a non-significant negative effect for crowding (dark grey bars).

Figure 4 compares the results from Experiments 1 and 2. Eliminating the illusory position shifts while leaving other stimulus attributes intact (such as speed, carrier spatial frequency, etc.), also eliminated the dependence of crowding on motion direction. The difference in crowding (Experiment 1) is not caused by motion per se, but rather by shifts in perceived position.

### Speed dependency

**Figure 5:**
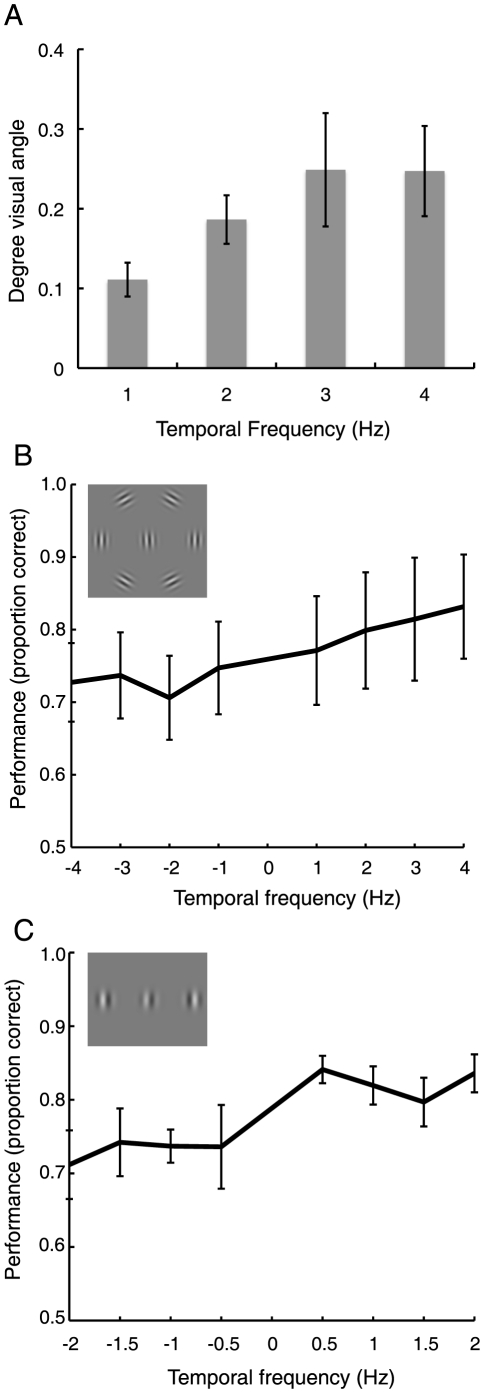
Effects of motion speed on mislocalization and crowding. **A** Change of PSEs in Experiment 3A as a function of drift speed (temporal frequency). Faster drift lead to a larger perceptual mislocalization (errorbars are between-participant standard errors). **B and C** Performance on the target discrimination task in Experiment 3B and C as a function of drift direction and temporal frequency. Changes to the stimuli in Experiment 3C are shown in the inset. Negative numbers on the x-axis denote inward drift towards the target, positive numbers outward drift away from the target (errorbars are between-participant standard errors).

We further evaluated whether changing the speed of motion in the distractors, which is known to modulate the perceived position shift [6], would also modulate the crowding effect. In Experiment 3A we sought to confirm that different drift speeds led to different magnitudes of perceived position shifts. Analogous to Experiment 1A, participants judged the perceived position of gratings drifting outward relative to inward. Because different temporal frequencies might alter the salience of the distractors relative to a static target, and because crowding is strongest when distractors are more similar to the target [2], the target was also made to drift away from the fixation point at the same frequency as the distractors. While this might lead to a perceived position shift of the target to a more eccentric position, the drift direction of the target remained constant for all directions and temporal frequencies of drift in the distractors. Therefore, any difference between inward and outward drifting distractors cannot be attributed to the fact that the target contained motion. Figure 5A shows mean PSEs for four participants. A repeated measures ANOVA confirmed that temporal drift frequency influenced the size of the perceived shift, *F*(3,9) = 9.87, *p* = 0.003. Higher drift speeds led to larger shifts in the perceived positions of drifting gratings.

In Experiment 3B, participants performed an orientation discrimination task on the central target. The physical positions of the distractors were fixed, only the drift speed and direction was varied across trials. Figure 5B shows performance as a function of drift speed and direction. A repeated-measures ANOVA confirmed that drift speed influenced the performance on target discrimination (*F*(7,21) = 11.73, *p*<0.001). Generally, faster speeds away from the target led to best performance, whereas faster speeds toward the target led to worse performance (linear regression, *r* ^2^ = 0.88, *p*<0.001). Experiment 3C replicated this result qualitatively with a different set of stimuli (Figure 5C). Again there was a significant effect of drift speed and direction on orientation discrimination (*F*(7,21) = 6.65, *p*<0.001); faster drift speeds away from the target led to better and faster drift speeds towards the target to worse performance (*r* ^2^ = 0.78, *p* = 0.002). 

## Discussion

In the present study we show that crowding is determined by the perceived position of objects more than their retinal position. When retinal and perceived positions are dissociated by an illusory motion-induced position shift [4]–[6], the amount of crowding is determined by the perceived positions of the flankers (Experiment 1). This is due to the perceived position shift and not due to some other aspect of the motion, since equivalent motion that does not cause a shift in perceived position does not lead to changes in crowding (Experiment 2). The change in crowding caused by the change in perceived position closely matches the expected change caused by a physical shift in position (Experiment 1). Modulating the perceived position shift by modulating the speed of drift also changes crowding in the expected way (Experiment 3).

### Potential influences of motion and grouping

The motion in the distractor Gabor patches might be expected to influence the visibility of the central target and thus performance on the orientation task. Facilitatory effects near the leading edge of a moving stimulus [6], [14]–[16] and inhibitory effects at the trailing edge [12], [13], [17]–[20] are well documented in the literature and may be causally contributing to motion-induced mislocalizations [5], [21]–[24]. However, our main finding is the opposite of what would be predicted from simple lateral facilitation or inhibition effects. Distractors drifting towards the target caused worse performance than distractors drifting away from the target (Experiment 1 and 3), but only when the motion also caused an illusory position shift of the distractors (Experiment 2). This is consistent with modulated crowding due to the perceived change in position of the distractors.

When distractors can be perceptually grouped, crowding influences on a target are diminished [25]–[27]. In the present stimulus, Gabor gratings were oriented tangentially forming an approximate “ring” around the target (see Figure 1), which might have reduced the overall magnitude of crowding. The tangential orientation was necessary to make distractors drift towards and away from the target. Nevertheless, there was sufficient crowding of the central target to measure a change in crowding dependent on the motion-induced mislocalization of distractors. In Experiment 3C we used a stimulus with just two distractors radially flanking the target, thus avoiding grouping, and found the same qualitative result (Fig. 5C).

### Implications for models of crowding

Our present study shows that crowding does not operate in strictly retinotopic coordinates, but takes into account the perceived position of objects. Our results agree with two previous reports, presented at recent conferences. Dakin and colleagues [28], [29] demonstrated that manipulating a target's perceived eccentricity and alignment with flankers modulated crowding, and Cavanagh and Holcombe [30] showed that crowding can be specific to the arrangement of distractor objects to a target object within a moving focus of attention (also see [2]). Here, crowding seems to work in an object-centered coordinate frame that moves with the locus of attention, when the object is attentively tracked. Together, the results suggest that visual motion is processed and locations (of objects and attention) are assigned before crowding occurs.

The mechanism for motion-induced changes of perceived position, such as the DeValois illusion used in the present experiments, is itself still debated. There are suggestions that lateral interactions in the feed-forward stream from the retina to V1 can explain position shifts in the neural activity profile on retinotopic maps [31], [32]. Other studies have provided strong evidence that feedback from extrastriate areas involved in motion processing is crucial for motion-induced mislocalizations, biasing activity at the higher-resolution map in V1 towards the direction of motion [33]–[36]. For example, the perceived location of a global motion stimulus is determined by the visual system only after integration of local motion signals into a global motion percept in higher areas is completed [37]. Recent evidence from neuroimaging shows that perceived object positions emerge in higher areas, while primary visual cortex representations remain more veridical to the retinal input [38]. If determining the perceived position of a moving grating or a moving object requires activity in higher areas and feedback, and visual crowding is determined by the perceived position, it follows that crowding itself cannot be determined in the feed-forward stream of visual processing.

Some recent findings have demonstrated that higher-level perceptual features can modulate the strength of crowding. For example, crowding is diminished when flankers can be grouped into a coherent Gestalt [25]–[27]. For face targets, crowding is increased when the flankers are also holistically perceived as faces [39], [40]. Another recent study demonstrated that when some distractors are rendered perceptually invisible, the number of perceived rather than physically present distractors determines the severity of crowding [41]. Consistently with our present finding, these studies demonstrate that the perceptual appearance of stimuli beyond their physical aspects influence crowding (reviewed in [42]). Our present finding, however, goes one important step further in showing that the spatial coordinate system itself in which crowding occurs, is not based purely on physical stimulus positions on the retina, but rather on perceived positions.

### Conclusion

Despite almost 100 papers on crowding published in just the last two years, no consensus about the underlying mechanism(s) has been reached [2], [42]. A dominant group of proposals suggests that crowding occurs when several objects fall within an integration field, a region of visual space where features are combined. Over-integration is thought to happen within one or more cortical retinotopic maps, implicitly reasoning that retinal distances between targets and flankers matter most [2], [43]. Some studies have shown that higher-level perceptual effects modulate crowding, but none have questioned the underlying spatial reference frame in which crowding occurs. Our results demonstrate that perceived distance determines crowding, suggesting that the putative over-integration occurs in a *perceptual* representation of space. Future investigations should not limit themselves to manipulations in purely retinotopic coordinates, and indeed, some basic defining characteristics of crowding (including critical spacing and Bouma's rule [3]) may require re-evaluation in terms of perceived positions.

## Materials and Methods

### Participants

Eight volunteers (four females and four males, age 20 to 29 years) participated in the present experiments. The study was approved by UC Davis Institutional Review Board; all participants gave written consent prior to the experiment. All had normal or corrected to normal visual acuity and were experienced psychophysical observers. Except for two authors, all other participants were naïve as to the hypotheses of the study. Five participants completed Experiment 1, three including one new participant Experiment 2, four including one new participant completed Experiment 3A and B, and four including one new participant Experiment 3C.

### Stimulus Presentation

Stimuli were presented on a 21″ Sony GPD520 CRT monitor at 100 Hz vertical refresh rate using MATLAB and the Psychophysics toolbox [44], [45]. Viewing was binocular; observers' heads rested on a chin and forehead rest at 54 cm viewing distance from the screen.

### Stimuli

Participants fixated a small white (90.0 cd/m^2^) circle on a mid-grey (30.1 cd/m^2^) background in the center of the screen. 14 degrees to the left or right side of the fixation mark a single static Gabor target was presented, a sine grating with 5.2 cycles per degree in a 2D Gaussian contrast envelope with a standard deviation of 1.6 degrees. The Gabor target was tilted from vertical by 2° in either clockwise or counterclockwise directions. The target was surrounded by six drifting Gabor distractors in a hexagonal arrangement (see Figure 1). Each distractor was oriented so that the drift direction (temporal frequency 4.2 Hz) on separate trials was either towards or away from the target (plus a random orientation jitter of +/− 2° to alleviate perceptual grouping of distractors). The presentation duration for each stimulus display was 500 ms (except in Experiment 3C, 1000 ms). In all experiments, left and right visual field stimuli were presented in alternating blocks (first block randomized between participants).

### Experiment 1

In Experiment 1A the stimulus was presented twice with 500 ms blank interval between presentations. In the first interval, distractors were placed at a distance 3.6 degree from the target and drifted either inward or outward (randomized between trials). In the second interval, drift direction was reversed and the physical position of the distractors was shifted between 0 and 0.6 degrees (in increments of 0.15 degrees) in the direction of motion in the first interval. Observers judged in which interval the Gabors appeared as spaced wider. The target in the center of the ring was present, but irrelevant for the task. Observers performed one block for each hemifield with 150 trials (2 initial motion directions × 5 physical position shifts × 15 repetitions).

In Experiment 1B, observers performed a 2AFC discrimination task on the target's orientation. Performance for both inward and outward drifting distractors was measured by varying the physical target-distractor distance between 1.9 and 5.1 degrees (in steps of 0.53 degrees). Observers performed one block for each visual hemifield with 360 trials (2 motion directions × 6 physical positions × 30 repetitions).

### Experiment 2

In Experiment 2 distractor gratings were presented with a hard aperture instead of a Gaussian contrast envelope and the luminance of the background was reduced to 0.13 cd/m^2^. All other aspects of the stimuli – including spatial and temporal frequency of the drifting gratings – and the task remained the same as in Experiment 1.

### Experiment 3

In Experiment 3A we used a two-interval forced-choice task analogous to Experiment 1A to measure the perceived shift of the distractor gratings for varying speeds. The temporal drift frequency was varied between 1 and 4 Hz in steps of 1 Hz, resulting in drift speeds between 0.2 and 0.8 degrees per second. (Note that we did not vary temporal and spatial frequency concurrently, so this experiment did not independently assess dependence of the effect on speed and temporal frequency.) In the first presentation interval, distractors were presented at 3 degrees distance from the target. In the second interval speed remained constant, drift direction was reversed, and positions were physically offset in the direction of motion in the first interval by between −0.2 to +0.6 degrees (in steps of 0.2 degrees). Participants judged whether the ring of distractors appeared as spaced wider in the first or second interval. Each participant performed one block for each visual hemifield with 200 trials (2 initial motion directions × 5 physical positions × 20 repetitions).

In Experiment 3B, analogous to Experiment 1B, participants judged the orientation of the central target grating, while the distractors drifted inward or outward at one of the four temporal frequencies (1 to 4 Hz). Distractors were always presented at 3 degrees distance from the target. The target drifted away from the fixation point at the same rates as the distractors. Participants performed the same 2AFC orientation judgment as in Experiment 1B. Each participant performed one block for each visual hemifield with 1024 trials (2 motion directions × 4 drift speeds × 128 repetitions).

In Experiment 3C only two distractors flanked the target radially (see inset of Figure 5C). The spatial frequency of the target and the distractors was 2 cycles per degree. The center-to-center distance between target and distractors was fixed at 3.5 degrees. Distractors were drifting at one of four temporal frequencies between 0.5 and 2 Hz towards or away from the target. The target remained stationary, but flickered in counterphase at a fixed frequency of 1 Hz. The presentation duration of the stimuli was extended from the previous experiments to 1 s.

### Analysis

Data from left and right hemifields of each observer were collapsed, as they were not significantly different (effect of hemifield on responses in Experiment 1A, *F*(1,4) = 0.45, *p* = 0.54). We also collapsed across trials with either motion direction in the first presentation interval (in Experiments 1A, 2A, 3A; effect of motion direction in first interval in Experiment 1A, *F*(1,4) = 1.67, *p* = 0.27). To measure the size of the mislocalization illusion and the influence of motion on crowding we estimated PSEs and 75%-correct thresholds by fitting cumulative Gaussian functions to individual observers' responses. Goodness of fit for all psychometric function fits was acceptable as assessed by the deviance statistic D [46] (D_Shift_Illusion_ = 3.64, SD = 3.65; D_Crowding_Inward_ = 4.99, SD = 3.41; D_Crowding_Outward_ = 4.23, SD = 2.18). 95%-confidence intervals were estimated with a parametric bootstrap procedure [47]. Inferential statistics on group data are reported in the text.

## Acknowledgments

The authors would like to thank Elvin Sheykhani for help with data collection.
